# Optimizing the Scale of a Wavelet-Based Method for the Detection of Gait Events from a Waist-Mounted Accelerometer under Different Walking Speeds

**DOI:** 10.3390/s19081869

**Published:** 2019-04-19

**Authors:** Carlotta Caramia, Cristiano De Marchis, Maurizio Schmid

**Affiliations:** Department of Engineering, Roma Tre University, via Vito Volterra 62, 00146 Rome, Italy; cristiano.demarchis@uniroma3.it (C.D.M.); maurizio.schmid@uniroma3.it (M.S.)

**Keywords:** initial contact, final contact, inertial sensor, wavelet, gait parameters

## Abstract

The accurate and reliable extraction of specific gait events from a single inertial sensor at waist level has been shown to be challenging. Among several techniques, a wavelet-based method for initial contact (IC) and final contact (FC) estimation was shown to be the most accurate in healthy subjects. In this study, we evaluated the sensitivity of events detection to the wavelet scale of the algorithm, when walking at different speeds, in order to optimize its selection. A single inertial sensor recorded the lumbar vertical acceleration of 20 subjects walking at three different self-selected speeds (slow, normal, and fast) in a motion analysis lab. The scale of the wavelet method was varied. ICs were generally accurately detected in a wide range of wavelet scales under all the walking speeds. FCs detection proved highly sensitive to scale choice. Different gait speeds required the selection of a different scale for accurate detection and timing, with the optimal scale being strongly correlated with subjects’ step frequency. The best speed-dependent scales of the algorithm led to highly accurate timing in the detection of IC (RMSE < 22 ms) and FC (RMSE < 25 ms) across all speeds. Our results pave the way for the optimal adaptive selection of scales in future applications using this algorithm.

## 1. Introduction

Wearable inertial sensors are being increasingly used to record human gait and quantify locomotion systems [1,2,3], when assessing the effects of aging [4], or when evaluating the effect of a cognitive [5] or a biomechanical load [6]. This increase in popularity stems from evidence that, while showing lower accuracy and robustness in the extraction of gait features than lab-based motion systems [7], inertial sensors reduce acquisition costs, widen the working area, allow long-duration analyses, and increase comfort during the set-up procedure [8]. A variety of methods have been thus introduced to increase reliability in the extraction of such gait features [9,10], and to reduce the number of wearable nodes to increase the impact of this technology for gait monitoring. If just one inertial sensor is to be used, placing it at the lumbar level has been shown to be a convenient and effective solution [11], since it makes it possible to gather information from both sides. It has been shown that step/stride time and its sub-phases, e.g., stance time, swing time, length and frequency, symmetry, regularity, and smoothness, are parameters that can be obtained from the lumbar-worn sensor with good accuracy [11,12].

The vast majority of the abovementioned spatiotemporal gait features first requires the individuation of the main gait phases (e.g., stride, stance, and swing phase) through detection of the timing of associated gait events, which are the initial contact (IC) and the final contact (FC). The IC is the time instant corresponding to when the foot first contacts the ground, while the FC occurs when the foot completely detaches from the ground. It is thus clear that an accurate and reliable estimation of these temporal events, taken bilaterally, is necessary for any successive application, such as the development of control algorithms for assistive devices in clinical scenarios (e.g., drop foot assistance and treatment [13]), or for investigating the harmony of gait [14].

Among the methods that involve the use of a single sensor unit located at waist level [15,16,17,18], the one proposed in [18] showed the most accurate estimates [11], with a mean absolute error of 20 ms for ICs and about 30 ms for FCs, when walking at a self-selected speed in a population of healthy individuals. The event estimation involved a wavelet-based filtering technique, applying the Gaussian Continuous Wavelet Transform (CWT) to smooth and differentiate the integrated vertical acceleration signal. Researchers showed that the use of wavelet transform was suitable for detection of gait events, allowing limitations of traditional techniques in smoothing simple and complex kinematic data [19,20] to be overcome.

The extent of filtering performed by the mother wavelet can be adjusted using a proper scale of the mother wavelet. McCamley and colleagues [18] set it to 16 for a 100 samples/s sampling rate (corresponding to a central frequency of 1.25 Hz), as it was shown to provide good results in terms of detection accuracy. A previous study of ours [21] showed the effect induced by the variation of the scale on IC and FC events estimation, when the gait speed was self-selected by healthy participants. It was found that the goodness of gait events estimation varied in response to a change of the scale value.

Thus, the aim of this study was to expand on the previously presented wavelet-based method through a systematic analysis evaluating the effects caused by the variation of the mother wavelet scale in the estimation of gait events at different walking speeds. This was done to check if a common optimal scale can be found, and to link it to variations in gait velocity and frequency.

## 2. Materials and Methods

### 2.1. Participants and Test Procedure

The experiment involved 20 young female students (age 24.8 ± 2.9 years, height 1.64 ± 0.05 m), without any reported motor disability or other problems which could influence the experiment. In order to exclude possible effects on the estimation due to reported differences in gait spatiotemporal parameters and gait pattern caused by sex differences [22,23,24], we opted to recruit a sample of only females. All of the participants provided a written informed consent in accordance with the Declaration of Helsinki. The research study was approved by the Ethics Committee of the Applied Electronics Section of the Department of Engineering (Ref. #01–018). They were asked to walk along a 6 m long straight path. The experiment consisted of three blocks, each one characterized by a different gait speed (i.e., normal, fast, and slow). Each block consisted of 10 repetitions of straight walking along the pathway. Each velocity was self-selected by the subject within each block. For each block, the first repetition was used to allow the subject to familiarize themselves with the self-selected velocity in the lab-space. For each repetition, only the two central strides were considered for the analysis, resulting in the detection of four IC events and three FC events. In some cases, when gait deceleration came early, only one central stride was considered, resulting in three ICs and two FCs.

### 2.2. Instrumentation

A magneto-inertial sensor (Shimmer3, Shimmer sensing, Dublin, Ireland) fixed at the waist level (L3) with an elastic belt was used to record accelerometer data (sampling rate 102.4 samples/s, range ± 2 g) and saved on an SD card for the offline procedure to detect IC and FC events. These estimations were then compared to the reference values obtained through a stereo-photogrammetric system (SMART DX 6000, BTS Bioengineering, Milan, Italy) which was equipped with eight cameras and used to capture markers placed on feet, following Davis’ protocol [25], at a sampling frequency of 250 samples/s. Reflective spherical markers (1.5 cm in diameter) were placed bilaterally on the heels, on the fifth metatarsophalangeal joints, and on the external lateral malleoli. The vertical acceleration was later resampled—with linear interpolation—at 250 Hz to match that of the stereo-photogrammetric system and obtain an event-by-event comparison with the same time resolution. The two systems were synchronized.

### 2.3. Data Processing

The reference gait events (IC_REF_ and FC_REF_) were obtained from the stereo-photogrammetric system (BTS Smart 6000) by tracking the trajectories of the markers on the heels and the fifth metatarsophalangeal joints. To estimate these events from the inertial sensor, we applied the method proposed in [18], which takes the vertical component of the acceleration, and uses a wavelet-based approach to filter data. In the original McCamley implementation, the acceleration is first integrated and then differentiated twice using the Gaussian CWT with a Gaussian mother wavelet of order one (corresponding to the first derivative of the Gaussian wavelet) and a scale of 16 at a sampling frequency of 100 samples/s. The locations of the minima of the smoothed acceleration signal obtained after the first differentiation identify the IC (IC_ACC_) timings, while the locations of the maxima of the smoothed jerk signal obtained after the second differentiation provide the FCs (FC_ACC_). This method showed a relative independence from the sensor alignment during the acquisition.

The filtering approach followed [26], and it is briefly covered in the following. In particular, given a signal *y*(t), the analytical formulation of its CWT *y_ω_*(*a*,τ) is as follows:

(1) $$y_{\omega }\left(a,\tau \right)=\;\int_{-\infty }^{+\infty }y\left(t\right)\frac{1}{\sqrt{a}}\Psi \left(\frac{t-\tau }{a}\right)dt$$

where *Ψ*(t) is the mother wavelet, *a* is an integer scaling parameter, and τ is the temporal shift.

When *Ψ*(t) has a fast decay, defined by the *m*-th derivative of a smoothing function with fast decay and a non-zero constant integral, the CWT is equal to a smoothed version of the signal derivative:

(2) $$\lim_{a\to 0}\frac{y_{\omega }\left(a,\tau \right)}{K_{s}^{3/2}}=\;\frac{dy\left(t\right)}{dt}$$

In this study, we implemented the previous method by using a Derivative of Gaussian (DoG) of order one mother wavelet as *Ψ*(t), which granted the fast decay condition. We then varied the wavelet scales in the range 1–100, which at 250 samples/s, corresponded to descending central frequencies from 50 Hz to 0.5 Hz. In this way, for each value of the scaling parameter, we had a time varying signal that could be further processed to extract IC_ACC_ and FC_ACC_ with the procedure previously described.

To compare the gait events estimates with the reference values, for each block of each subject, and by using all the scales in the previously mentioned scale range, we calculated the temporal differences between the reference value taken from the motion capture system and the values coming from the wavelet-based procedure for each IC and FC event. If the absolute value of this difference was lower than a specified tolerance range, the closest event within this tolerance range was considered as a true positive (TP), and the corresponding difference was extracted. Possible additional events in the same window were considered as false positives (FP), while a false negative (FN) was added if no event was found within that tolerance range. The tolerance range was set at 50 ms according to typical error values reported in the literature [11] (slightly higher than the maximum value of the mean absolute estimation error). Then, sensitivity as TP/(TP + FN), precision as TP/(TP + FP), and *F*1_score_ as 2 * sensitivity * precision/(sensitivity + precision) were calculated for the overall sample.

For each TP, the root mean square of the temporal difference value vector (RMSE) was then calculated, and its value normalized to the tolerance range, to obtain *RMSE_N_*. We then defined a cost function *T*, following the formulation introduced by [27], which combines the *F*1_score_ and *RMSE_N_*, according to the equation below:

(3) $$T=1-\left(1-RMSE_{N}\right)*F1_{score}$$

*T* was then used as a unique compact cost index to identify the best scale (Scale_opt_) for each velocity of each subject. In particular, Scale_opt_ was identified as the scale corresponding to the absolute minimum of *T* as a function of scales.

Thus, for each scale of the wavelet-based approach we had four performance/cost parameters (sensitivity, precision, *F*1_score_, and RMSE) and a compact cost index, *T*. This was repeated for the different speed conditions of each subject. To check for relations between the cost index function of the wavelet scale and biomechanical factors, both the gait speed and step frequency of each trial were extracted.

A linear regression analysis was then conducted between Scale_opt_ and step frequency across subjects and speeds to test the prediction power of step frequency on algorithm parameters (i.e., the mother wavelet scale). The significance level was set at 0.05.

## 3. Results

Subjects performed the different bouts with speeds equal to 0.84 ± 0.11 m/s, 1.14 ± 0.15 m/s, and 1.56 ± 0.17 m/s, for the slow, normal, and fast self-selected speeds, respectively. We observed a strong correlation between gait speed and step frequency (R^2^ = 0.92), which was equal to 1.54 ± 0.16 s^−1^, 1.87 ± 0.16 s^−1^, and 2.29 ± 0.17 s^−1^, for the slow, normal, and fast self-selected speeds, respectively.

**Figure 1:**
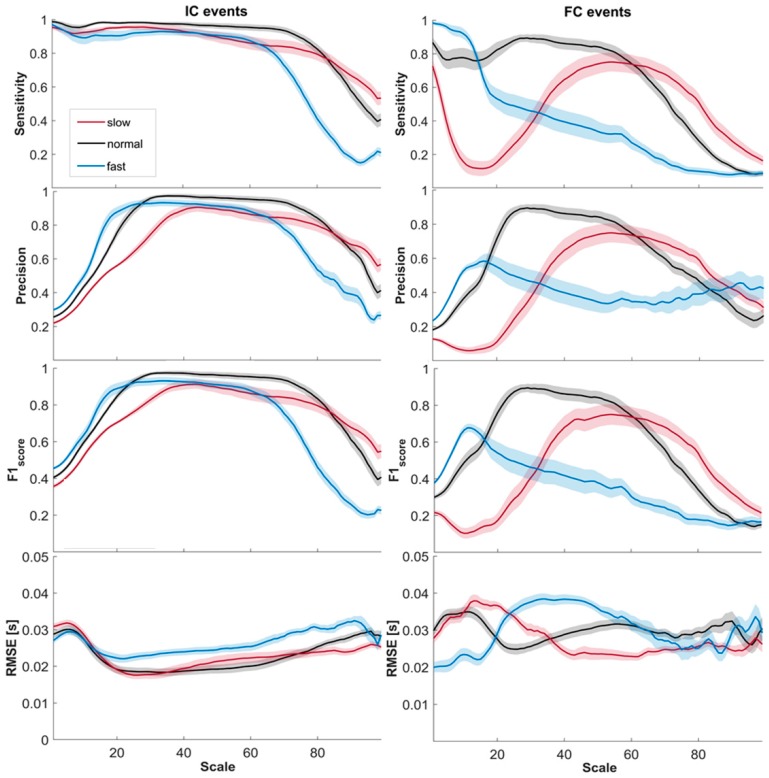
Means ± standard errors of sensitivity, precision, *F*1_score_, and RMSE values obtained for the slow, normal, and fast speeds for the initial contact (IC) events (**on the left**) and for the final contact (FC) events (**on the right**).

Figure 1 shows the mean values (colored lines) ± the standard errors across subjects for the four parameters previously introduced, for both the IC events and the FC events, as a function of the scale value, while also considering the three gait speeds separately.

Considering the IC events (left panels of Figure 1), the error in the estimation was limited for a wide range of scales. Sensitivity was high for scales up to 60, while precision decreased for scales lower than 10 and higher than 70, due to a non-negligible number of false positives. The *F*1_score_ showed values higher than 0.9 for scales in the range 38–49 for slow speed, 23–74 for normal speed, and 22–55 for fast speed. The common range of scales with an *F*1_score_ value higher than 0.9 was thus 38–49 (corresponding to central frequencies in the range 1.024–1.3158). The minimum RMSE was found at scale 26 (18 ms) for slow speed, at scale 26 (19 ms) for normal speed, and at scale 22 (22 ms) for fast speed.

Considering the FC events (right panels of Figure 1), only normal speed reached the *F*1_score_ value of 0.9 at scale 29. At slow speed it reached a maximum value of approximately 0.75 at scale 54, while at fast speed it was around 0.68 at scale 11. The same can be seen for both sensitivity and precision. The minimum RMSE was found at scale 61 (23 ms) for slow speed, at scale 26 (25 ms) for normal speed, and at scale 1 (20 ms) for fast speed (however, in the latter case we had an *F*1_score_ of only 0.38).

**Figure 2:**
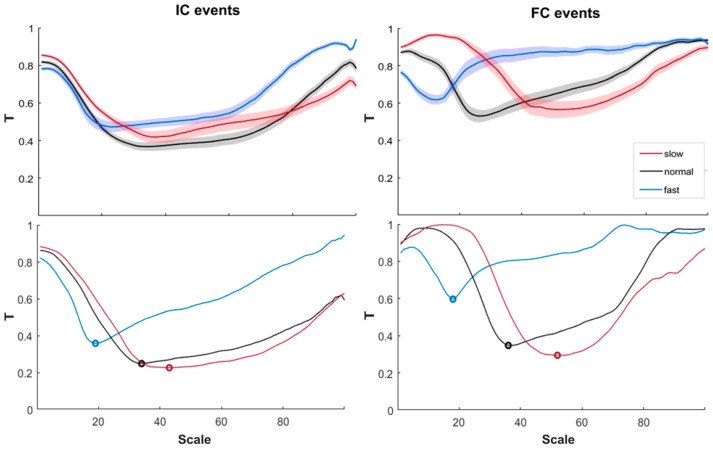
Upper panels: means ± standard errors of cost function *T* obtained for the slow, normal, and fast speeds for the IC events (**on the left**) and for the FC events (**on the right**). Bottom panels: a representative example of *T* for one subject. Circles indicate the minima of *T* for the three speeds for both gait events.

The upper panels of Figure 2 show the mean values ± the standard errors across subjects of the cost function *T* for the IC events and the FC events, while the bottom panels show the same for one subject taken as an example, to highlight the subject specificity of the optimal scale.

**Figure 3:**
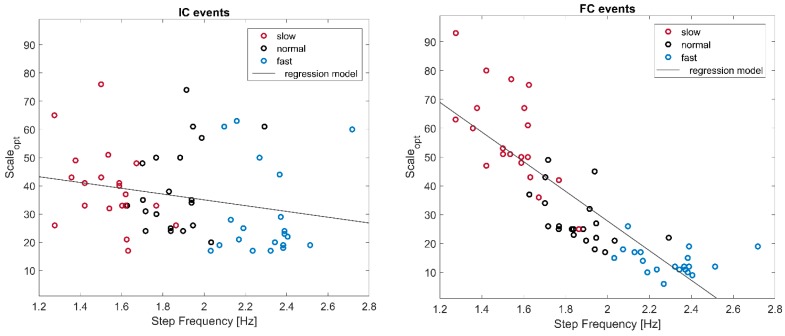
Representation of the optimal scale value in relation to step frequency for the three gait speeds. Each empty circle refers to one subject at a chosen speed, and grey lines represent the linear regression models (the relationship between variables).

In Figure 3, the values of Scale_opt_ (corresponding to the minimum of the compact cost function *T*) are represented against step frequencies (*SF*). Since we found a very high correlation between the normalized (to subject’s height) gait speed and the corresponding step frequency (*p* = 8.7 × 10^−33^; pairwise linear correlation = 0.96), we considered only the step frequency as a possible predictor of Scale_opt_.

A negative correlation between Scale_opt_ and SF was present for ICs (*p* = 0.08; pairwise linear correlation = −0.23), and very marked for FCs (*p* = 1.1 × 10^−17^; pairwise linear correlation coefficient = −0.85). The corresponding regression analysis for IC events yielded the following linear model:

(4) $$Scale_{opt}^{IC}=-10*SF+56$$

while, for FCs:

(5) $$Scale_{opt}^{IC}=-10*SF+56$$

where *SF* is expressed in steps/s.

The same model can be expressed in terms of wavelet central frequencies (thus, independent from the chosen sampling frequency) as follows:

(6) $$f_{opt}^{IC}=0.69*SF+0.34$$

(7) $$f_{opt}^{FC}=3.6*SF-4.5$$

## 4. Discussion

The results of this work highlighted the presence of variability in events estimation goodness depending on the chosen mother wavelet scale. This dependence was significantly more evident for the estimation of final contacts with respect to initial contacts. The variability also depended on the subject’s gait speed/step frequency. In this sense, a functional relationship between the optimal scale and the step frequency was inferred.

Regarding the gait events estimation performance, while the scale choice may have played a marginal role for the detection of initial contact events, it showed a strong effect on the estimation of the final contacts.

Considering initial contact events, self-selected normal speed led to a very large range of scales for which performance was good, while for fast and slow speeds, the location of the optimal ranges shifted towards lower and higher scales, respectively, with a common reduction of the scale range for which the detection performance was high.

Goodness in final contacts detection was lower overall for all the gait speeds, and the ranges of scales for which the error was limited were narrower (especially for fast speed). Moreover, the tendency of the optimal scale range to shift to larger scales at slow speeds, and in the other direction for fast speeds, was even more pronounced than for initial contacts. Results were much better at high scales for low speed, at central scales for normal speed, and at low scales for fast speed.

Regarding the comparison of the detection of timing errors with respect to the literature, at the pseudo-frequency of 1.25 Hz—which corresponds to scale 16 for a sampling rate of 100 samples/s, as in [18], and to scale 40 for the sampling rate chosen in this study—mean absolute differences were found to be slightly lower than those found in [18]. Specifically, we obtained an error of 16 ± 5 ms for the initial contacts estimates and an error of 26 ± 7 ms for final contacts estimates. These values became even lower when selecting the optimal scales for the normal self-selected speed (15 ± 5 ms and 21 ± 7 ms, with central frequencies of 1.852 Hz and 2 Hz, respectively).

When using a general descriptor of performance, including both accuracy and timing characteristics, the existence of a relation between the step frequency and the optimal scale was explored. We used step frequency instead of gait speed, because the former can be directly obtained from the spectra of accelerometer data in an easy and accurate way [28], while the latter needs additional processing steps for its calculation [16,29]. The presence of a strong correlation between step frequency and gait speed in our sample size further supports our choice.

**Figure 4:**
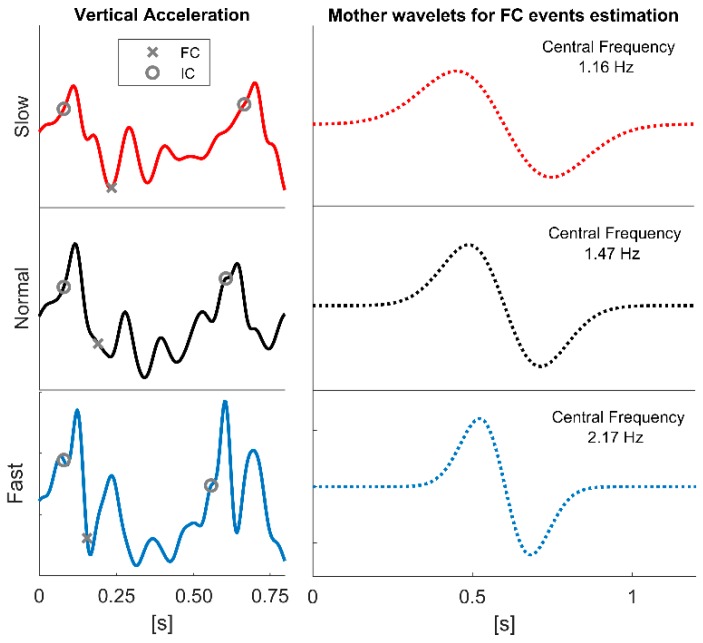
Sample data of vertical accelerations corresponding to one step for the three speed blocks (left panel). The reference initial and final contacts are indicated with a circle and with a cross, respectively. On the right, the corresponding mother wavelets that return the best estimation performance for final contact events are shown (the corresponding central frequencies are also reported).

From the analysis performed on the optimal scale, we determined two regression models, one for the initial contacts (Equation (4)) and one for the final contacts (Equation (5)). A clear relation between optimal scale and step frequency was found for the final contacts, while for the initial contacts this relation was at the significance threshold limits. Thus, the identification of the optimal scale is less dependent on the step frequency for the initial contacts, and this is paralleled by evidence showing that many scales lead to low estimation errors, and arethus suitable in terms of accuracy. On the contrary, in the case of the final contacts, a high level of regression was found, and this allowed to identify the best scale based on the step frequency. We speculate that a higher step frequency will lead to a higher frequency content of the acceleration patterns (as they occur over a narrower time scale, see Figure 4). Accordingly, the best frequency to smooth the pattern needs to be adjusted (i.e., increased), together with the corresponding wavelet scale (i.e., decreased). This may also apply to initial contacts, but in this case, the presence of a pattern that is more easily identifiable [11] makes the choice not crucial.

In terms of study limitations, these regression models were obtained using data coming from a sample population of female subjects. It may be interesting to evaluate whether the relation between the optimal scale and the step frequency is still valid for a sample of male individuals. If not, it may indeed be necessary to make an adjustment to the model, such as adopting a weighting scheme, in order to generalize the model. As a matter of fact, males and females adopt different walking strategies due to their anatomical differences, which determine the differences in gait pattern [22,23,24].

Regarding the shape of the mother wavelet used, the choice of the Derivative of Gaussian of order one was used following the original method. However, given the rather peculiar patterns associated with the different gait phases, it may be worth evaluating how the use of alternative mother wavelets influences the events estimation. This could help in choosing a mother wavelet which better matches the shape of the acceleration peak when a gait event occurs. This aspect may be of use when dealing with gait impairments, where alterations in accelerometer patterns lead to inaccuracies in the initial and final contact estimations [30].

The will to optimize the internal parameter of this wavelet-based method [18]—which already introduced an enhancement in the estimates of gait events for normal speeds compared to other traditional methods [11]—lies in the advantages of the method itself. Indeed, it requires a single component of acceleration as an input, without even needing an accurate placement and alignment of the sensor. This method exploits the characteristics of the wavelet transform [31] by removing the unwanted peaks from the acceleration signals and preserving the timing of peaks corresponding to the temporal events [18]. As a result, the instrumentation cost is reduced, and the setup is simplified.

The presence of a clear relation between the estimation of the final contacts and the step frequency opens up an interesting scenario, where an adaptive approach for the detection of final contacts can be designed. By calculating the step frequency from two successive initial contacts and using the regression equation, one can choose the optimal scale on a step-by-step basis to overcome decreases in accuracy caused by variations in step frequency (or gait speed) and step-time variability.

In this work, we considered a sample that was limited to young females. This choice was made to exclude the potential effects of sex, age, and anthropometric differences [3,32,33], even though we did not expect a significant effect on the identified regression model. However, further investigation involving subjects of different sex, ages, and anthropometry may be needed in order to obtain a model that includes these factors, for the determination of the optimal scale based on the adopted step frequency. Moreover, for further validation of this method, individuals with motor disorders could be included, since the identification of specific gait events could be more challenging [30]. Finally, we tested our optimized wavelet-based method on walking signals acquired while walking on flat straight paths, but additional validation could be gained from walking on irregular surfaces.
